# Hyper-speed meaning and form predictions: An EEG-based representational similarity analysis

**DOI:** 10.3758/s13423-025-02731-4

**Published:** 2025-07-25

**Authors:** Armando Quetzalcóatl Angulo-Chavira, Alejandra Mitzi Castellón-Flores, Haydee Carrasco-Ortiz, Natalia Arias-Trejo

**Affiliations:** 1 Facultad de Psicología, Universidad Nacional Autónoma de México, C-10, Av. Universidad 3004, Col. Copilco Universidad, Delegación Coyoacán, C.P. 04510 CdMx, México; 2 Facultad de Lenguas y Letras, Universidad Autónoma de Querétaro, Querétaro, México

**Keywords:** Prediction, Form, Meaning, Electroencephalography, Representational similarity analysis

## Abstract

Language comprehension involves predictive processing, in which comprehenders anticipate both semantic and form-related attributes of upcoming words. This predictive mechanism is crucial as it enables efficient real-time language processing, allowing listeners and readers to keep pace with rapid information streams and quickly correct potential errors. Using electroencephalography (EEG) and representational similarity analysis (RSA), we investigated whether predictions follow a hierarchical, top-down process or occur in parallel, facilitated by associative mechanisms. In this study, native Spanish-speaking undergraduate students read highly constrained sentences designed to elicit specific target words. RSA was applied to evaluate the similarity between all possible pairs of expected words, and signals were classified into semantic, form-related, or specific-word effects based on their relationship to the expected word. The results revealed a rapid transition between effects, with semantic predictions consistently preceding form-related and specific-word predictions. While the sequential order aligns with hierarchical processing, this rapid predictive transition is better understood in the context of associative mechanisms and predictive coding.

## Introduction

Language comprehension involves the incremental and dynamic integration of visual (printed words) or auditory (speech) information, enabling the inference of communicative intent. This process entails mapping sounds to meanings and can be facilitated by the prediction of upcoming linguistic elements (Kuperberg & Jaeger, 2016; Pickering & Gambi, 2018; Pickering & Garrod, 2013).

Based on this foundation, there is evidence supporting that comprehenders use sentence context to predict both the semantic and the form representations of words under certain conditions (Angulo-Chavira et al., 2022; Arias-Trejo et al., 2013, 2019; DeLong et al., 2005, 2019; Dikker & Pylkkänen, 2013; Dikker et al., 2009, 2010; Gagnepain et al., 2012; Gambi et al., 2018; Ito, 2019; Ito et al., 2016; Kim & Lai, 2012; Kukona, 2020; Kukona et al., 2014, 2016; Laszlo & Federmeier, 2009; Li et al., 2022; Martin et al., 2018; Mornati et al., 2023; Otten & Van Berkum, 2008; Otten et al., 2007; Pickering & Garrod, 2013; Sohoglu et al., 2012; Van Berkum et al., 2005; Wang et al., 2020, 2024; Wei et al., 2023; Wicha et al., 2004).^1^ For example, Wang et al. (2020) recorded EEG signals while presenting sentences constrained to either animate or inanimate words. They found increased similarity in the spatio-temporal pattern of electrical activity for animate compared to inanimate sentence pairs before the presentation of the expected word. This indicates that individuals can predict the animacy of expected words, suggesting they retrieve part of the semantic features of words. Likewise, Wei et al. (2023) evaluated the prediction of Chinese idioms using a similar approach. They compared words that shared the same last syllable with those that had different syllables. The results showed increased EEG signal similarity when the word ended with the same syllable, suggesting that participants predictively recovered part of the expected word form.

Most research on predictive language processing has concentrated on demonstrating that prediction involves the preactivation of words and meanings. Far less research has focused on understanding the mechanisms behind anticipatory language processing (Huettig, 2015; Ryskin & Nieuwland, 2023). According to the predictive coding framework, predictions in language comprehension are based on a top-down hierarchical mechanism (Kuperberg & Jaeger, 2016; Ryskin & Nieuwland, 2023), in which higher-level cognitive processes – such as sentence pragmatics, meaning, and syntax – constrain lower-level processes, including the retrieval of specific word representations (Kuperberg & Jaeger, 2016). In this framework, prediction follows Bayesian inference to generate parallel, weighted predictions based on context. This process involves mapping across hierarchical representations: the semantic level maps onto the lexical level, which, in turn, maps onto the form level. These predictions are continuously compared with lower-level input to update their weights.

Production-based frameworks (e.g., Pickering & Gambi, 2018; Pickering & Garrod, 2013) propose that the prediction of specific words also follows a hierarchical process. In this view, the production system^2^ is inherently structured across various linguistic levels, including semantics, syntax, and phonology, and plays a crucial role by offering a scaffold for making anticipatory predictions about upcoming words. Consequently, these frameworks suggest that lexical prediction during language comprehension follows the production path: higher-level representations, such as word semantics, are retrieved first and subsequently constrain lower-level predictions, such as form representation.

Hierarchical models predict, for example, that in the sentence, “In the airport, I board the airplane,” the context can activate semantic features such as function (boardability) and category (transportation). This semantic preactivation then triggers the activation of the word form, including the graphemes/A-I-R-P-L-A-N-E/or the phonemes/ɛrpleɪn/. Crucially, only the most relevant representation, given the context, is predicted.

Alternatively, it is possible that during lexical prediction, both types of information are recovered in parallel during language prediction via an associative mechanism (Huettig, 2015; Pickering & Gambi, 2018; Pickering & Garrod, 2013). This mechanism relies on the strength of associations built from previous experiences and exposure to language. From this perspective, linguistic representations can activate networks of related representations, such as semantic or form-related ones, with activation flow not restricted to any particular direction (Huettig, 2015). For example, in a sentence like “In the airport, I board the airplane,” the word “airport” can activate “airplane” due to their semantic and form-related connections. Importantly, other irrelevant predictions, such as “pilot” or “bird,” may also arise because these words are associated with “airplane” within the sentence context.

Although previous research has shed light on the temporality of retrieving semantic and form representations during prediction (DeLong et al., 2021; Heilbron et al., 2022; Ito et al., 2016), to the best of our knowledge, only Wang et al.’s (2024) study has provided evidence of hierarchical processing during prediction. They presented highly constrained sentences while recording EEG signals. Sentence pairs were designed to elicit predictions for either semantically related words (e.g., bank [financial] – loan) or form-related words (e.g., homographs: bank [financial – river]), before the onset of the expected target words. Wang et al. (2024) reported a semantic effect from –391 to –309 ms and a form effect from –53 to –8 ms (times relative to target onset). The authors interpret this result as evidence of hierarchical processing, where higher levels inform lower levels during prediction.

Although Wang et al. (2024) offer solid results, they are inconclusive. Their findings could be explained by differences in the processing times of homographs. Since homographs share form representation, they are processed more slowly than non-homographs (e.g., Duffy et al., 1988; Martin et al., 2024). It is also possible that these effects may reflect post-lexical activation driven by the recovery of the expected word rather than the activation of semantic and form features produced by the sentence context.

Previous research has demonstrated that predicting a word can trigger the activation of related information (Kukona, 2020). For instance, hearing the word “dentist” might lead to the prediction of the word “mouth.” However, this prediction can also activate phonologically similar words, such as “mouse.” Thus, the results reported by Wang et al. (2024) may be explained by the activation of semantically related concepts and homographs as a consequence of predicting the expected word within the specific sentence context. Indeed, previous work by the same group (Wang et al., 2018) estimated that the retrieval of a specific word occurs earlier (− 880 to − 485 ms) than the activation of their semantic and form effects.^3^

The present research aimed to contribute to the debate on hierarchical versus parallel mechanisms of prediction by measuring the temporality of semantic and form attributes relative to the prediction of a specific word. We conducted a representational similarity analysis (RSA) on the EEG signals recorded as participants read highly constrained sentences. RSA assumes that mental or neural representations can be characterized by similarity structures – meaning that representations of different stimuli or concepts can be described by their pattern similarities or dissimilarities (Kriegeskorte & Kievit, 2013; Kriegeskorte et al., 2008). Although the analysis is agnostic about neural organization, recent research has demonstrated that concepts are represented by the activation of specific neural groups, with related objects activating only a subset of neurons within the same group (Quiroga, 2012). These neuronal groups can be distributed across the entire brain (Huth et al., 2016; Pulvermüller, 2001). Consequently, when RSA is implemented, neural patterns for the same or a related stimulus should be more similar to each other than to those of different or unrelated stimuli (Kriegeskorte & Kievit, 2013; Kriegeskorte et al., 2008).

Thus, our experimental task consisted of pairs of highly constrained sentences designed to elicit the prediction of the same final word (e.g., The cat was being chased by the *dog*/I bought bones at the pet store for my *dog*). We hypothesized that the neural patterns preceding the presentation of an expected word would be more similar when sentences predict the same word (e.g., dog–dog) compared to when they predict different words (e.g., dog-piano), indicating a word-specific effect. Additionally, we calculated representational similarity across all possible sentence combinations. We further hypothesized that sentences predicting semantically related words (e.g., dog-cat) or form-related words (e.g., dog-doll) would produce more similar brain patterns than those predicting unrelated words (e.g., dog-truck), indicating semantic and form-based predictions. Consequently, if prediction operates hierarchically, neural similarity effects would appear sequentially: first the semantic prediction, followed by the form prediction, and finally the specific word prediction. However, if predictive retrieval occurs in parallel, all effects would manifest almost simultaneously.

## Method

### Participants

The sample comprised 31 undergraduate students from the Autonomous University of Queretaro (UAQ), with an average age of 22.45 years (*SD* = 4.20), and included eight males. These participants were all native Spanish speakers, right-handed, and reported having normal or corrected-to-normal vision and normal hearing. An additional six participants were excluded from the analysis due to experimental errors (*n* = 2) or high levels of artifact contamination (*n* = 4). The sample size of 24 exceeded the number (*n* = 17) determined necessary by our power analysis, which assumed a medium effect size for a single paired comparison (*δ* = 0.73, *α* = 0.05, *β* = 0.80), based on Wang et al.’s (2018) supplementary data. This study received approval from the Ethics Committee of the National Autonomous University of Mexico (Approval number: FPCE_ 21012021_H_AC) and adhered to the principles of the Declaration of Helsinki for experimental procedures involving human participants. All participants were asked to sign a consent form before participating in the study.

### Stimuli

In this study, we selected 94 highly constrained sentences from the sentence-final completion norms for Mexican Spanish (Angulo-Chavira et al., 2023). Selection was based on the final word of each sentence and its cloze probability. We chose 47 highly representative and concrete nouns, each linked to two highly constrained sentences (94 sentences). These 47 target words were selected to ensure significant semantic and orthographic relatedness variability across all possible pairings. Notably, while pairs of sentences were designed to predict the same word, we carefully selected contexts with different themes to ensure that observed differences in brain activity were attributable to the prediction of the upcoming word rather than the processing of similar contexts. To ensure uniform sentence length throughout the experiment, all sentences were standardized to eight words by adding an adverbial phrase or modifying the syntactic structure while preserving similar semantic content. Additionally, we replaced the penultimate word in each sentence with a syntactically plausible yet unexpected word. Moreover, we developed 47 yes/no questions related to the sentences. Although these questions were designed to be straightforward, answering them accurately required participants’ full attention.

### Validation studies

Four validation studies were conducted to assess sentence plausibility, the cloze probability of both the final and penultimate words, and the reliability of comprehension questions. These studies were carried out online via the Cognition platform (https://www.cognition.run/). To participate, individuals were required to agree to an informed consent form; without this agreement, they were unable to proceed with the task. Each participant was assigned to only one pilot study and did not take part in the main experiment.

#### Plausibility

This pilot study aimed to assess the plausibility of sentences among Mexican Spanish speakers, as any anomalies in semantic or syntactic structures could lead to incorrect prediction signals, potentially affecting semantic and phonological predictions for the critical word. The study involved 100 undergraduate students (51 males and 49 females) with an average age of 23.13 years (*SD* = 3.50, range** = **18–29 years). Participants rated the plausibility of sentences on an analog scale from 0 to 1, where zero indicated unnatural sentences and one indicated natural sentences. The results showed an average plausibility rating of 0.90 (*SD = *0.60). One-sample Wilcoxon signed-rank tests comparing these ratings to the chance level (0.5) indicated that all sentences were rated significantly above chance (all** p-**values < 0.001), demonstrating that participants found the sentences to be plausible.

#### Cloze probability of the final word

While cloze probability was already normed in the original corpus (Angulo-Chavira et al., 2023), the changes made to the sentences could influence their predictability. Consequently, the set of 94 sentences was evaluated by 101 undergraduate students (*M*_*age*_ = 23.21 years, *SD* = 3.41; 25 males) using a cloze probability procedure (Taylor, 1953). The results showed that the sentences exhibited high cloze probability (*M* = 0.93, *SD* = 0.09). This is highly important because we expected participants to activate a specific word, including its semantic and form information.

#### Cloze probability of the prefinal word

While the predictability of the final word is crucial, it is possible that observed electrophysiological differences during the epoch of the prefinal word could be attributed to the prediction of the prefinal word itself, rather than the final word. The set of 94 sentences underwent a cloze probability task in which the sentence context was presented without the final and prefinal words; participants were asked to provide the most appropriate continuation to the sentence. The task involved 100 university students (*M*_*age*_ = 23.21 years, *SD = *3.41; 25 males). The results indicated that the prefinal words had a low cloze probability (*M* = 0.04, *SD* = 0.07), suggesting that participants could not reliably predict the prefinal word.

#### Question validation

The pilot study aimed to determine whether participants had sufficient time to answer questions and whether the questions were straightforward enough to yield high accuracy. In this task, each of the 47 sentences was displayed for 4,000 ms, followed by its corresponding question for another 4,000 ms. Participants had to assess whether the question was congruent with the preceding sentence. This pilot study involved 70 university students (*M*_*age*_ = 22.32 years, *SD* = 3.41; 34 males). The results indicated a high percentage of correct responses (*M* = 98.38%, *SD* = 2.15), suggesting that the task was easy enough for participants to perform with high accuracy. On average, participants responded in 1,558.16 ms (*SD = *57.70), though some needed more time (up to 2,200 ms in some trials). Based on these findings, we adjusted the response time window to 2,400 ms.

### Experimental design

**Fig. 1:**
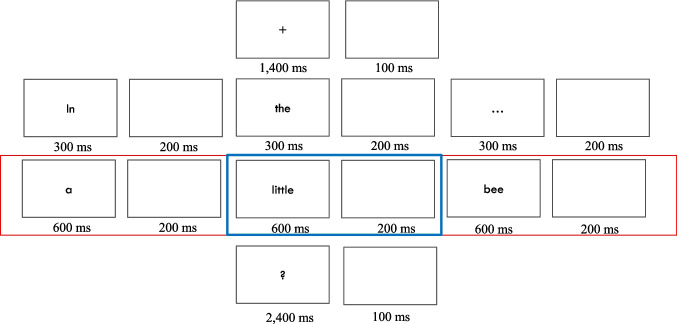
Example of an experimental trial. The stimuli were presented in a sequential format, moving from left to right and from top to bottom on the screen. The red rectangle in the display timeline indicates the phase during which the last three words of each sentence were presented at a slower pace. The blue rectangle highlights the analysis window, aligned to the presentation of the prefinal word. The question mark denotes the presentation of a question; however, in half of the trials, participants were shown a sequence of numeral symbols (######) for a duration of 900 ms instead

In the main experiment, the 94 sentences were displayed in a single block, with each word centrally positioned on the screen in black font against a gray background. The word height was adjusted to 1.5° of visual angle to ensure readability. The sequence began with a fixation point visible for 1,400 ms, followed by an interstimulus interval (ISI) of 100 ms (Fig. 1). The first five words of each sentence were then presented for 400 ms each, separated by ISIs of 100 ms. The presentation of the last three words differed; they were shown for 600 ms each, with ISIs extended to 200 ms. Following the sentence display, participants were either shown a string of numeral symbols (#####) for 900 ms or a yes/no question for 2,400 ms, each followed by an ISI of 100 ms.

This adjustment in the presentation rate of words – transitioning from a faster to a slower pace – was designed not only to shorten the overall experiment duration but also to enhance the detection of representational similarity increases previously documented by Wang et al. (2018).

### Data acquisition

The electroencephalographic (EEG) signal was captured using a NuAmps amplifier with a sampling rate of 1,000 Hz. The setup included 34 silver/silver-chloride electrodes, positioned according to the international 10–10 system layout (Fp1, Fp2, F7, F3, Fz, F4, F8, FT7, FC5, FC3, FCz, FC4, FC6, FT8, T7, C3, Cz, C4, T8, TP7, CP5, CP3, CPz, CP4, CP6, TP8, P7, P3, Pz, P4, P8, O1, Oz, O2). Additionally, five extra electrodes were utilized: one served as the grounding electrode, two were placed on the earlobes for re-referencing purposes, and the remaining two were to monitor vertical and horizontal eye movements. The impedance levels of all electrodes stayed below 10 kΩ.

Data collection was conducted using Curry 7 software, while the behavioral tasks were presented through Stim2 software. Both systems were synchronized using a Cedrus Stim Tracker Quad. Participants’ responses were collected via a Cedrus response pad, model RB-740.

### Procedure

Upon arrival at the laboratory, participants followed a standard sanitary protocol to maintain a clean and safe environment. They were then asked to sign an informed consent form and provide demographic information. This was followed by preparations for the electroencephalographic recording. Participants were seated approximately 60 cm away from the screen and were instructed to use the index and middle fingers of their right hand to interact with the response pad during the experimental tasks. Specifically, participants were asked to press the green button for questions that were congruent with the previously presented sentence and the red button for incongruent questions. For tasks involving the presentation of numeral symbols, participants were instructed not to respond.

To confirm that all instructions were clearly understood, a brief familiarization phase consisting of three trials was conducted at the beginning of the experiment. If a participant responded incorrectly during this phase, the experimenter provided feedback, and the familiarization trials were repeated as needed. Throughout the experiment, participants were observed and monitored via a Gesell chamber, allowing the experimenter to ensure that instructions were followed correctly and to provide corrections when necessary.

### Data processing

The preprocessing of the EEG data was conducted using EEGLAB (Delorme & Makeig, 2004). The procedure began with re-referencing the electroencephalographic signal to the average of the signals from the earlobes. This was followed by the removal of the direct current (DC) component to ensure the baseline stability of the signal. The artifact treatment consisted of four stages to maximize data quality for analysis. The first stage involved the removal of line noise at 60 Hz. In the second stage, a fourth-order Butterworth bandpass filter with a range of 0.5–30 Hz was applied to isolate the frequency band of interest. The third stage focused on automatic artifact correction. This involved the use of the “runica” algorithm combined with the *ICAlabels* extension for EEGLAB to identify and remove independent components associated with muscle activity, eye movements, heart signals, and channel noise. Finally, an artifact subspace reconstruction process was implemented to correct or reject residual artifacts not fully addressed by the previous steps. The EEG signal was segmented into epochs extending from − 800 ms to 0 ms relative to the expected onset of the final word, with a focus on the period associated with the presentation of the prefinal word.

#### Temporal similarity

To investigate the temporal dynamics of lexical prediction during language comprehension, we employed RSA, a method that quantifies the similarity in patterns of neural activity elicited by linguistic stimuli (Kriegeskorte & Kievit, 2013; Kriegeskorte et al., 2008). Figure 1 shows a scheme of the RSA computation. We computed RSA by correlating the patterns of neural responses across the scalp, captured through EEG recordings. For RSA, we extracted the neural activity patterns from these epochs and performed pair-wise comparisons using Pearson’s correlation coefficient. This resulted in similarity time series for each participant, reflecting the degree of similarity in neural responses to the compared stimuli. With 94 sentences included in our study, this resulted in a total of 4,371 unique sentence pairs.

After calculating the representational similarity time series, we adapted the regression-based ERP method (Smith & Kutas, 2015a, b) to our signals to estimate RSA (rRSA) while accounting for the relationship between sentence pairs using three regressors: Word-Specific, Semantic Similarity, and Form Similarity.

The Word-Specific regressor was based on the matching of identical words using a dummy-coded scheme. If the expected final words of the sentence pairs used to compute the RSA were the same, the regressor was coded as 1, indicating a Within-Word correlation; if they were different, it was coded as 0, indicating a Between-Word correlation.

The Semantic predictor was a continuous variable, computed using the WordNet corpus and Lin’s similarity measure to assess semantic relationships between words (Lin, 1998). Lin’s similarity, which ranges from 0 to 1, quantifies the degree of semantic similarity between two words, with 0 indicating no similarity and 1 denoting high similarity. This measure is based on the information content of the least common subsumer (the most specific ancestor node they share) in a taxonomy, such as WordNet. The informational content is calculated from the SemCor corpus, reflecting how often words appear in specific contexts. For our study, Lin’s similarity was calculated for all 4,371 pairs of target words to assess their semantic closeness.

The Form predictor was a continuous variable that assessed orthographic similarity and was calculated using the normalized edit similarity measure (Yujian & Bo, 2007). This similarity metric quantifies the number of substitutions, additions, and deletions required to change one string of characters into another, with its values ranging from 0 to 1. A score of 0 indicates no orthographic similarity between two words, whereas a score of 1 signifies high similarity.

The rRSA was conducted independently for each participant to model individual brain activity during the prediction period. It involved multiple linear regression models across time, implemented in Python using the *ordinary least squares* function from the *statsmodels* package. Variance inflation factor analysis confirmed that collinearity among the three predictors was within an acceptable range (Word-Specific: 3.79, Semantic: 1.30, Form: 3.57), ensuring reliable model estimation (Salmerón et al., 2018). Accordingly, we modeled the RSA signal as follows:

$$RSA \approx {\beta }_{0}+ {\beta }_{1}(Word-Specific)+ {\beta }_{2}(Semantic) + {\beta }_{3}(Form) + \varepsilon$$

To ensure a straightforward statistical analysis,^4^ we computed the rRSAs under four different conditions as follows:

$$\begin{array}{c}{Word-Specific}_{rRSA}\approx {\beta }_{0}+{\beta }_{WS}\left(1\right)+{\beta }_{Sem}\left(1\right)+{\beta }_{Form}\left(1\right)+\varepsilon \\ {Semantic}_{rRSA}\approx {\beta }_{0}+{\beta }_{WS}\left(0\right)+{\beta }_{Sem}\left(1\right)+{\beta }_{Form}\left(0\right)+\varepsilon \\ \begin{array}{c}{Form}_{rRSA}\approx {\beta }_{0}+{\beta }_{WS}\left(0\right)+{\beta }_{Sem}\left(0\right)+{\beta }_{Form}\left(1\right)+\varepsilon \\ {Unrelated}_{rRSA}\approx {\beta }_{0}+{\beta }_{WS}\left(0\right)+{\beta }_{Sem}\left(0\right)+{\beta }_{Form}\left(0\right)+\varepsilon \end{array}\end{array}$$

In this framework, the Word-Specific rRSA represents the predicted signal with the highest similarity, as identical words share the maximum value (1) across all three predictors. Conversely, the Unrelated rRSA represents the predicted signal with the lowest similarity, where all predictors are set to 0. The Semantic and Form rRSA signals isolate the influence of their respective predictors by setting them to 1 while keeping the other two predictors at 0.

#### Cross-temporal similarity

It is important to note that our hypothesis regarding different time courses for distinct types of predictions implies that these predictions arise from different neural generators. If different temporal windows truly reflect distinct processing stages, there should be no generalization across these time windows.

To test this hypothesis, we conducted a cross-temporal generalization analysis (King & Dehaene, 2014) by correlating the spatial pattern vector of a given sentence (*S*_*i*_, where *i* = 1 to 94) at a specific time (*t*_*k*_, where *k* = −800 to 0) with all the time samples (*t*_*k*_ from *k* = −800 to 0) of another sentence (*S*_*j*_, where *j* = 1 to 94 and *i ≠ j*). Due to the high computational cost of this analysis, we downsampled the EEG signal from 1,000 Hz to 300 Hz, resulting in 4,371 cross-temporal similarity (CTS) matrices, each measuring 240 × 240.

Following a procedure similar to the RSA analysis, we used a regression-based approach to estimate individual predicted signals (rCTS) for each participant. Using the same regressors (Word-Specific, Semantic, and Form), we computed four rCTS: Word-Specific, Semantic, Form, and Unrelated.

### Statistical analysis

#### Temporal similarity

The dependent variables for the temporal analysis were the estimated rRSA time series across subjects in the four conditions (Word-Specific, Semantic, Form, and Unrelated), specifically within the prediction window, spanning from − 800 to 0 ms relative to the anticipated onset of the target word. This time window aligned with the presentation of the penultimate word (Wang et al., 2018, 2024). Initially, the analysis window was defined using a one-sample *t*-test, comparing the representational similarity time series against 0.02, which corresponded to the median value of the average across the four conditions.

**Fig. 2:**
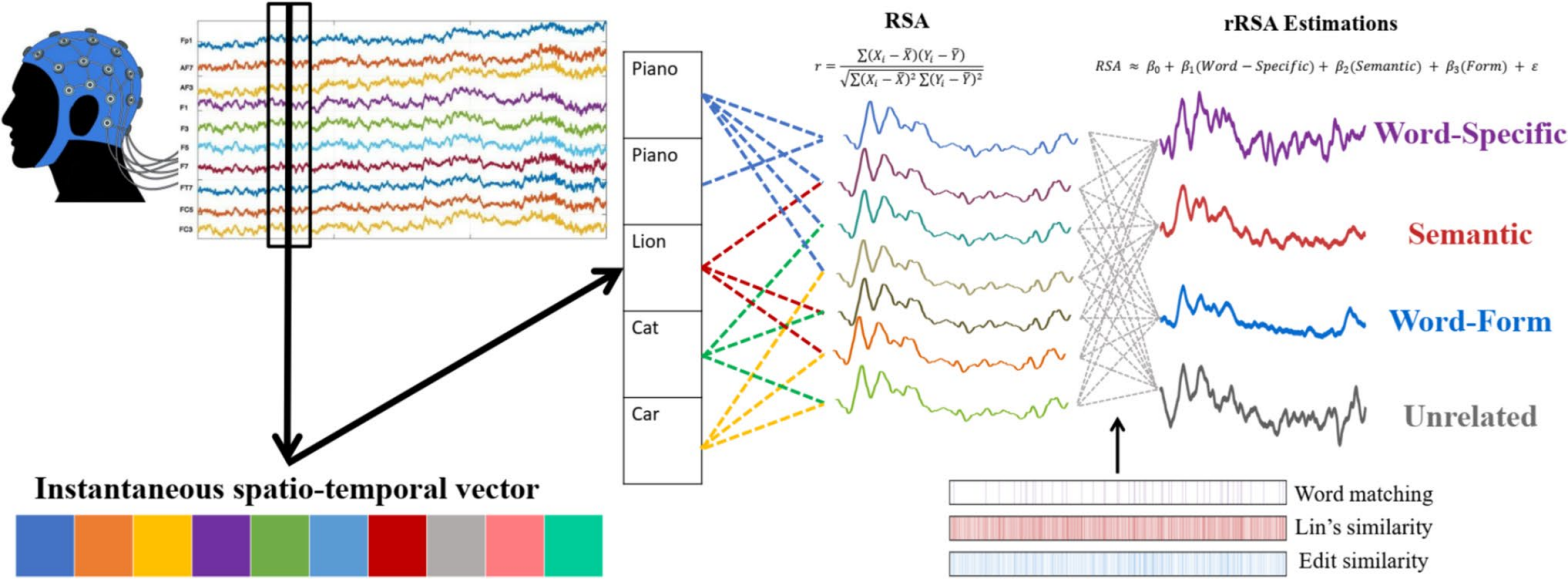
Procedure used in the regression-based representational similarity estimation

A comparison was considered significant if it remained so after applying the Benjamini and Yekutieli (2001) multiple comparison correction. Notably, three clusters of consecutive significant values were identified within the analysis window, indicating periods of heightened representational similarity: − 730 to − 687 ms, − 658 to − 579 ms, and − 560 to − 511 ms. Since these clusters represent peaks within the main increase (Fig. 2), we defined an analysis window that encompassed all three clusters of significant values. Thus, the first analysis window ranged from − 730 ms to − 511 ms.

**Fig. 3:**
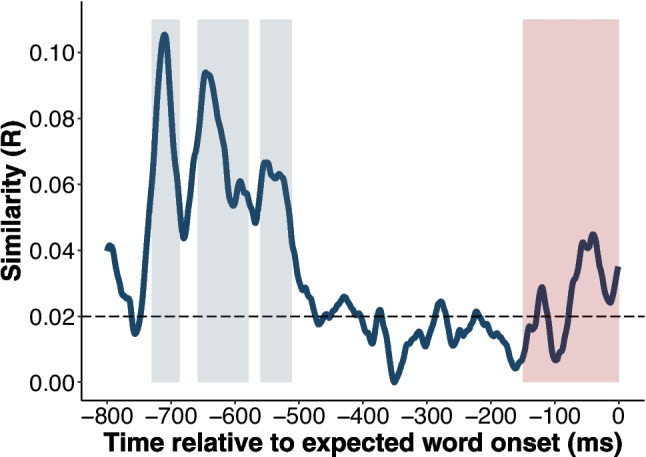
Average of all representational similarity time series. The plotted line displays the average of all the representational similarity signals. The horizontal dashed line represents the chance level (0.02). The gray-shaded region highlights the significant differences against chance level

As shown in Fig. 3, there was also an increase in similarity at the end of the trial. Although this increase was not significant, we defined a second analysis window based on previous findings suggesting that phonological effects emerge near the presentation of the final word (Wang et al., 2024). Accordingly, this second analysis window ranged from − 150 to 0 ms.

The formal analysis was conducted in two steps. First, a cluster permutation analysis (Maris & Oostenveld, 2007) was performed to determine whether predictive effects were present in the data. Second, the cluster analysis was repeated using a jack-knife resampling procedure to explore the latency of the effects. In both analyses, the primary comparisons contrasted the Word-Specific, Semantic, and Form rRSA signals against the Unrelated rRSA signal.

In all the cluster analyses, the formation of clusters involved summing adjacent t-values that surpassed 2.04, corresponding to an alpha level of 0.05 (two-tailed). To create null distribution, maximal clusters were identified from 100,000 permutations (Pernet et al., 2015), in which signals were randomly shuffled between conditions. A cluster reached significance if its aggregated value exceeded 95% of all cluster values within the null distribution.

Although cluster permutation analysis is highly effective for detecting effects while controlling for multiple comparisons, it does not allow precise inferences about the timing of effects. This limitation arises because the analysis tests entire clusters rather than specific time points (Sassenhagen & Draschkow, 2019) and because the onset and duration of effects depend on the chosen threshold and permutation procedure (Ito & Knoeferle, 2023).

To address this, we used cluster permutation analysis as a mask (Sassenhagen & Draschkow, 2019) in the second analysis to measure the latency of the maximum *t*-value within the largest cluster. Importantly, we did not infer temporality directly from the cluster analysis; instead, we used it to estimate latency within a cluster defined by a reasonable threshold (*t* = 2.04). In each iteration of the jack-knife resampling procedure, we extracted a latency value, which was then analyzed using a permutation test with 100,000 randomizations. The *p*-values were computed by comparing the permuted distribution of latency values with the observed *t*-value, using an alpha level of 0.05. Latency comparisons were conducted only when the effect was present in the cluster analysis.

#### Cross-temporal similarity

For the cross-temporal analysis, the dependent variables were the estimated rCTS matrices across subjects independently for the four conditions (Word-Specific, Semantic, Form, and Unrelated). These were analyzed within the prediction window from − 800 to 0 ms relative to the anticipated onset of the target word. The primary goal of this analysis was to determine whether temporal activity within each condition exhibited generalization across time windows. To test this, we applied cluster permutation analysis to the entire trial.

Clusters were formed by summing adjacent *t*-values exceeding 2.04, corresponding to an alpha level of 0.05 (two-tailed), with comparisons made against 0. The null distribution was generated by identifying the largest clusters across 100,000 permutations, during which signals were randomly shuffled between conditions. A cluster was considered significant if its summed *t*-value exceeded the 95th percentile of the null distribution. Since temporal generalization could result in multiple significant clusters, we applied a Bonferroni correction to maintain the overall error rate at 0.05%.

## Results

### Behavioral

Participants exhibited strong performance on the task, achieving an average accuracy of 94.31% (*SD* = 0.58). These results indicate that the participants were highly attentive and actively engaged in the behavioral task.

### Temporal similarity

After artifact rejection, an average of 85.52 epochs (*SD* = 14.89) per participant remained for analysis.

**Fig. 4:**
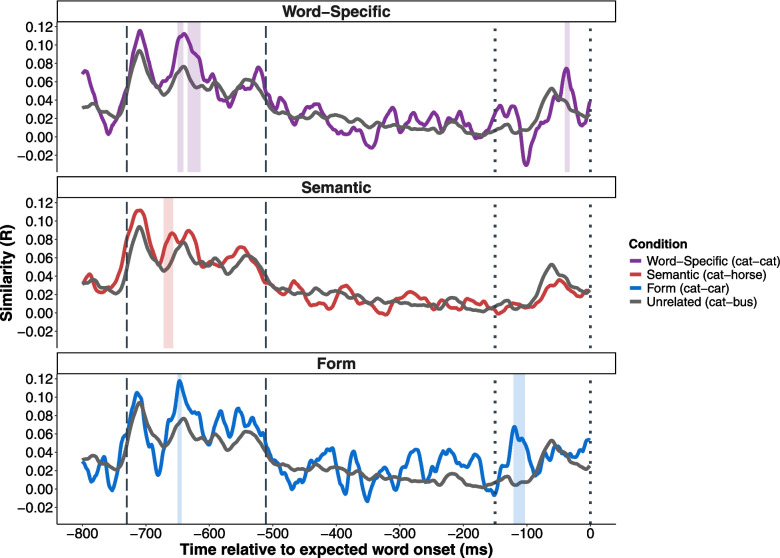
Representational similarity time series. Time series of the average estimated representational similarity for the Word-Specific (purple), Semantic (red), and Form (blue) conditions, with the Unrelated condition (gray) included as a baseline comparison. The x-axis represents time in milliseconds relative to the expected word onset. The dashed and dotted vertical lines mark the boundaries of the analysis windows. Shaded regions indicate statistically significant clusters where similarity values differ significantly from the baseline condition

In the first analysis window, two significant clusters were identified, where similarity in the Word-Specific condition was higher than in the Unrelated condition. These effects occurred at around − 650 to − 641 ms (*t*_*cluster*_ = 16.89, *t*_*max*_ = 2.17, *p* < 0.001) and − 634 to − 614 ms (*t*_*cluster*_ = 46.72, *t*_*max*_ = 3.01, *p* < 0.001), respectively (Fig, 4, upper panel). Similarly, the Semantic similarity signal was significantly higher than in the Unrelated condition (*t*_*cluster*_ = 43.08, *t*_*max*_ = 3.74, *p* < 0.001), occurring at around − 672 to − 657 ms (Fig. 4, middle panel). Finally, there was a significant increase in representational similarity in the Form condition compared to the Unrelated condition (*t*_*cluster*_ = 11.26, *t*_*max*_ = 2.34, *p* < 0.001), with this effect occurring at around − 650 to − 644 ms (Fig. 4, bottom panel). The jack-knife^5^ resampling procedure showed that the Semantic effect (− 662 ms) emerged significantly earlier than both the Form (− 642 ms) and Word-Specific (− 632 ms) effects, occurring ~ 16 ms before the Form effect (*t*(29) = 5.66, *p* < 0.001) and ~ 30 ms before the Word-Specific effect (*t*(29) = 12.47, *p* < 0.001). Additionally, the Form effect preceded the Word-Specific effect by ~ 14 ms (*t*(29) = 197.11, *p* < 0.001).

In the second analysis window, we found a significant increase in Word-Specific similarity compared to the Unrelated condition (*t*_*cluster*_ = 13.36, *t*_*max*_ = 2.35, *p* < 0.001), with this cluster extending from − 40 to − 33 ms (Fig. 4, upper panel). In this window, there was no significant difference between the Semantic and Unrelated representational similarity signals (Fig. 4, middle panel). However, Form similarity was significantly higher than Unrelated similarity (*t*_*cluster*_ = 39.39, *t*_*max*_ = 2.42, *p* < 0.001) in a cluster extending from − 121 to − 103 ms (Fig. 4, bottom panel). The jack-knife^6^ resampling procedure showed that the Form effect (− 110 ms) occurred significantly earlier than the Word-Specific (− 38 ms) effects with a gap of ~ 20 ms (*t*(29) = 20.46, *p* < 0.001).

### Cross-temporal similarity

The cluster permutation analysis revealed significant increases in similarity across all four conditions when compared to zero (Word-Specific: *t*_*cluster*_ = 1,634, *t*_*max*_ = 6.88, *p* < 0.0001; Semantic: *t*_*cluster*_ = 3,734, *t*_*max*_ = 7.12, *p* < 0.0001; early Form: *t*_*cluster*_ = 879, *t*_*max*_ = 5.48, *p* = 0.0004; late Form: *t*_*cluster*_ = 958, *t*_*max*_ = 5.23, *p* = 0.00001; Unrelated: *t*_*cluster*_ = 4,104, *t*_*max*_ = 7.52, *p* < 0.0001).

**Fig. 5:**
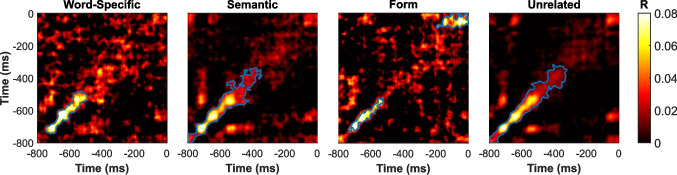
Cross-temporal analysis. Heatmaps representing the average Cross-Temporal similarity analysis estimated from the regression models: Word-Specific, Semantic, Form, and Unrelated. The x-axis and y-axis indicate time in milliseconds (ms) relative to stimulus onset. The color scale represents the estimated correlation values (R), with warmer colors indicating higher similarity values. Significant clusters are outlined in blue

At a descriptive level, these increases appear near the diagonal, with some variation in cluster extension. However, they largely overlap with the first analysis window in the temporal similarity analysis: Word-Specific, − 746 to − 485 ms; Semantic, − 800 to − 341 ms; Form, − 734 to − 522 ms; and, Unrelated, − 800 to − 319. Notably, only the Form condition exhibited a second cluster at the end of the trial (around − 143 to 0), which also aligns with the late analysis window in the temporal similarity analysis Fig. 5).

## Discussion

A central question in language comprehension is how the brain predicts upcoming information. Lexical prediction involves anticipating words based on contextual cues and retrieving their semantic and form representations. Our study tested two hypotheses: the hierarchical mechanism, where semantic predictions precede form predictions, and the parallel mechanism, where both are retrieved simultaneously (Kuperberg & Jaeger, 2016; Pickering & Gambi, 2018). To investigate these mechanisms, we presented participants with highly constrained sentences that elicited the prediction of specific target words while recording their brain activity using EEG. We analyzed these signals using regression-based RSA (Kriegeskorte & Kievit, 2013; Kriegeskorte et al., 2008) to compare neural patterns for semantic, form-related, and word-specific predictions against unrelated ones.

In our approach, a rise in representational similarity indicates shared neural activation during prediction. When semantic RSA increases, the brain’s spatial patterns overlap with other semantically related words (e.g., “dog” and “cat” show greater similarity than “dog” and “car”). On the other hand, an increase in form similarity reflects orthographic overlap (e.g., “car” and “cat” exhibit more similarity than unrelated words). Finally, identical words display the highest similarity among all possible combinations.

Our results revealed that semantic effects emerged before form effects, which preceded word-specific effects during an early period, from around − 670 to − 640 ms. In a later period, from around − 120 to − 30 ms, form effects preceded word-specific effects but not semantic effects. A cross-temporal similarity analysis (King & Dehaene, 2014) suggested that the representational effects were transient, reflecting independent stages driven by different neural generators.

Our findings further support the retrieval of meaning and form representations during lexical prediction in language comprehension. This retrieval involves not only the preactivation of specific word representations but may also generate the preactivation of features from other meaning and form representations that share similarities with the activated concepts. These activations appear to originate from specific neural generators that produce transient, time-dependent activations (King & Dehaene, 2014).

More importantly, from a theoretical perspective, our results align with the expected temporality predicted by hierarchical frameworks (Kuperberg & Jaeger, 2016; Pickering & Gambi, 2018; Ryskin & Nieuwland, 2023), in which high-level representations (e.g., semantics) are retrieved before lower-level representations (e.g., word form).

Although our results provide congruent evidence of hierarchical processing in language prediction, the time lag suggests that this process can occur more rapidly than previously reported (Wang et al., 2024). Our findings reveal a temporal difference of approximately 16 ms between semantic (− 642 ms) and form effects (− 632 ms). In contrast, Wang et al. (2024) reported a 300-ms gap (semantic: − 367 ms; form: − 25 ms), which they interpreted as evidence of hierarchical processing based on a prediction-by-production mechanism.

According to production-based frameworks, language comprehension relies on predictive mechanisms implemented by the production system (Dell & Chang, 2014; Martin et al., 2018; Pickering & Gambi, 2018; Pickering & Garrod, 2013). Consequently, prediction should follow the same temporal dynamics as speech production (~ 600 ms), except for the articulatory stage. Based on this assumption, the temporal gap between semantic and form processing would range between 150 and 400 ms (Indefrey & Levelt, 2004), aligning with the findings of Wang et al. (2024).

One possible explanation for the difference in time lag between semantic and form predictions in our data compared to Wang et al. (2024) may be methodological. Specifically, the timing estimates by Wang et al. (2024) could be overestimated due to their use of homographs as proxies for word forms. Homographs typically require longer processing times, which could extend to prediction processes as well (Azuma et al., 2004; Burke et al., 2004; Martin et al., 2024).

Nevertheless, we also observed a word-form effect (− 110 ms) and a word-specific effect (− 38 ms) at the end of the trial. However, only the word-form effect was evident in the cross-temporal analysis (from − 200 to 0 ms). It is possible that Wang et al. (2024) captured only the late stage of prediction, a possibility they also acknowledged in their paper. Thus, our results replicate Wang et al.’s (2024) findings while extending them by demonstrating distinct stages in the prediction process that involve the activation of form representations.

These rapid transitions between semantic and word-form preactivations seem better explained by associative mechanisms or predictive coding than by the discrete-stage “prediction-by-production” framework proposed by Pickering and Gambi (2018). Associative mechanisms do not imply a strictly sequential pattern, yet they explain the rapid transition in our findings. Dell (2013) suggests that predictions based on production could emerge from cascading activation and feedback. Therefore, semantic processing does not need to be completed before phonological processing begins (Dell, 1986), which would increase processing speed.

In this cascading model, the timing of semantic and form preactivations depends on activation thresholds. For example, in “I bought bones at the pet store for my dog,” the word “dog” receives more semantic than phonological activation from “bones” and “pet”; phonological activation occurs in parallel but to a lesser degree. However, when form-related words like “dig” or “dodge” also boost the phonological representation of “dog,” both semantic and phonological representations may emerge simultaneously. Thus, a strictly sequential pattern may mainly appear in highly constrained contexts, where one or more words strongly prime the target. Consequently, multiple lexical candidates are partially activated at once until the predictive system accumulates enough evidence to select the most likely word. This framework accounts for the rapid transition from semantic to form representations but does not explain why word-specific effects emerge after semantic and form effects.

Alternatively, our data can also be explained by predictive coding (Kuperberg & Jaeger, 2016; Ryskin & Nieuwland, 2023). This framework does not assume the involvement of the production system but similarly posits that semantic information is activated before word-form representations. Brothers et al. (2023) argued that multiple representations are preactivated based on probabilistic inferences derived from sentence context. Notably, these preactivations are continuously updated as incoming information is compared with lower-level representations. As information is updated, certain lexical candidates are selected while those that are no longer relevant are ruled out.

Consequently, in our experiment, it is possible that the predictive system initially evaluates multiple semantic competitors that share neural representations. These representations are then mapped onto specific lexical and word-form representations. However, once all the evidence has been integrated, alternative candidates at both the semantic and word levels are discarded, leaving only the most probable word-specific prediction. This explains why word-specific effects emerge after semantic and word-form effects rather than encompassing both simultaneously.

A crucial question arising from these findings is why prediction appears biphasic – an early phase involving semantic, word-form, and word-specific predictions, and a later phase restricted primarily to word-form and possibly word-specific activations. Pickering and Gambi (2018) describe two distinct predictive stages: prediction-by-association and prediction-by-production. The initial stage, prediction-by-association, is rapid, automatic, and unspecific, driven entirely by word associations. Notably, this stage is undirected – activated words relate to the processed word rather than necessarily to the upcoming word. In contrast, the prediction-by-production stage integrates multiple cues, such as visual context and shared background knowledge, to predict the most probable upcoming word. Semantic, syntactic, and form representations are explicitly predicted through production mechanisms, provided there is sufficient time and cognitive resources. Otherwise, partial predictions are made (e.g., only semantic), or predictions are omitted entirely.

Support for dual-stage prediction comes from Corps et al. (2022), who demonstrated distinct semantic and gender-based prediction stages. Participants listened to male or female speakers produce sentences such as “I would like to wear the nice…” while being presented with semantically related targets (e.g., tie, dress) and distractors (e.g., drill, hairdryer). Initially, participants looked at both semantically related targets upon hearing the verb but later narrowed their focus based on speaker gender cues. Corps et al. (2022) interpreted these results as evidence of an initial associative stage followed by a second stage that selectively used contextually relevant information.

Our findings partly support a two-stage prediction framework. During the early prediction stage (with a 16-ms gap), the timing of shifts between semantic, form, and word-specific effects can be interpreted as arising from associative mechanisms. However, in contrast to Pickering and Gambi (2018) and Corps et al. (2022), our early-stage predictions appear to be specific; that is, they preactivate the upcoming input rather than broadly activating concepts. Indeed, both semantic and form effects were clearly aimed at the next word since the RSA was computed based on the similarities of the expected targets. In the later stage, semantic aspects seem to be resolved, leaving the predictive system to refine form-level predictions. One possibility is that the production system drives the slower preactivation of form, as Pickering and Gambi (2018) propose. In this view, prediction-by-production mainly refines form representations rather than semantic ones.

In contrast, from the perspective of predictive coding, this biphasic behavior can be explained by the updating of predictions. Although the exact speed of prediction and updating processes remains unclear, it must be rapid enough to support real-time language comprehension. However, unlike spreading activation models – where activation propagates freely – predictive coding does not impose the same temporal pressure on the updating process (Brothers et al., 2023). In highly constrained sentences, semantic information likely generates less uncertainty than word-form information, requiring greater verification for word-form representations. Furthermore, this verification process may be triggered by the predictability of the expected word, which might not occur in natural reading. Given that typical reading speeds are approximately 249 words per minute (Brysbaert, 2019), participants could read around three words within our 800-ms time window. This suggests that the second prediction stage could be optional or adaptive. However, in our experimental design, this verification process may have been facilitated by the predictability of the final word. Thus, when participants anticipate that the next word is about to appear, they engage in predictive verification or reinforcement of the prediction.

## Considerations

We primarily examined the sequence of semantic and form predictions, which can be explained by either a cascading associative production model or predictive coding. However, our current method does not allow us to disentangle these frameworks. Future research measuring how semantic information modulates form processing could provide further insight into this issue.

Additionally, we focused on highly constrained sentences, which are effective for isolating semantic and form retrieval processes. Investigating sentences with lower constraint, however, could help reveal how different predictive mechanisms influence the observed temporal dynamics.

## Conclusion

Our findings reveal that semantic preactivations precede form and word-specific representations, supporting an rapid yet hierarchical process of lexical prediction. The brief gap between semantic and form effects suggests that these predictions may rely on a rapid mechanism, as proposed by associative and predictive coding frameworks. While our results are consistent with both a cascading associative production model and predictive coding, future research employing less constrained contexts will be necessary to disentangle these frameworks and provide further insight into how meaning and form interact during real-time language comprehension.

## Data Availability

The material, data, and scripts that support the findings of this study are available at the Open Science Framework at: https://osf.io/qye3a/?view_only=2f3ddd92da2d4a13bba37d216900050c

## Footnotes

1 Although there is convincing evidence for the prediction of word form, some studies have failed to find word-form prediction under specific conditions, such as certain experimental methodologies (Ito et al., 2016; Nieuwland et al., 2018) or populations (Ito, 2019; Ito & Sakai, 2021; Ito et al., 2018).

2 Note that the association of the production system with prediction during language comprehension has been supported by theoretical, computational, experimental, and neuropsychological evidence (Dell & Chang, 2014; Martin et al., 2018; Pickering & Gambi, 2018; Pickering & Garrod, 2013).

3 We must acknowledge that the difference could also be explained by the method, as Wang et al. (2018) used a slower rate of presentation than Wang et al. (2024). Even with the current evidence, we cannot rule out the possibility of a post-predictive effect.

4 Note that we cannot compare the β values directly across dummy-coded (Word-Specific) predictors and continuous predictors (Semantic and Form), as their interpretations differ. While the β value for a dummy-coded predictor (Word-Specific) represents the difference in RSA between the reference category (Word-Specific = 0) and the category coded as 1 (Word-Specific = 1), the β values for continuous predictors (Semantic and Form) indicate the expected change in RSA for a one-unit increase in the respective predictor.

5 The conclusion drawn from the jack-knife analysis remains the same whether we use the highest *t*-value in the analysis period or the highest *t*-value within the maximum cluster (Semantic vs. Word-Specific: *t*(29) = 378.58, *p* < 0.001; Semantic vs. Form: *t*(29) = 6.72, *p* < 0.001: Form vs. Word-Specific: *t*(29) = 6.41, *p* < 0.001). The only difference is that the latency of the Word-Specific cluster is estimated slightly later (− 618 ms) in the former approach.

6 The conclusion drawn from the jack-knife analysis remains the same whether we use the highest *t*-value in the analysis period or the highest *t*-value within the maximum cluster (Form vs. Word-Specific: *t*(29) = 20.40, *p* < 0.001).
